# The Role of Mast Cells in IgE-Independent Lung Diseases

**DOI:** 10.1007/s12016-020-08779-5

**Published:** 2020-02-21

**Authors:** Daniel Elieh Ali Komi, Esmaeil Mortaz, Saeede Amani, Angelica Tiotiu, Gert Folkerts, Ian M Adcock

**Affiliations:** 1 Immunology Research Center, Tabriz University of Medical Sciences, Tabriz, Iran; 2 Department of Immunology, Tabriz University of Medical Sciences, Tabriz, Iran; 3 Clinical Tuberculosis and Epidemiology Research Center, National Research Institute of Tuberculosis and Lung Diseases, Shahid Beheshti University of Medical Sciences, Tehran, Iran; 4 Division of Pharmacology, Utrecht Institute for Pharmaceutical Sciences, Faculty of Science, Utrecht University, Utrecht, The Netherlands; 5 Respiratory Section, National Heart and Lung Institute, Faculty of Medicine, Imperial College London, London, UK

**Keywords:** Mast cells, Non-IgE mast cell activation, Antigen-independent, COPD, IPF, Lung cancer

## Abstract

Mast cells (MCs) are granular cells of the innate immune system which develop from CD34^+^/CD117^+^ progenitors and play a role in orchestrating adaptive immune responses. They have a well-known role in allergic reactions following immunoglobulin (Ig)E-mediated activation of the cell-surface expressed IgE high-affinity receptor (FcεRI). MCs can also respond to various other stimuli due to the expression of a variety of receptors including toll-like receptors (TLRs), immunoglobulin (IgG) receptors (FcγR), complement receptors such as C5a (CD88) expressed by skin MCs, neuropeptides receptors including nerve growth factor receptor, (NGFR), cytokines receptors such as (IL)-1R and IL-3R, and chemokines receptors including CCR-1 and CCR-3. MCs release three groups of mediators upon degranulation differentiated according to their chemical composition, storage, and time to release. These include preformed mediators (mainly histamine, tryptase, and chymase), de novo synthesized mediators such as prostaglandin (PG)D2, leukotriene (LT)B4 and LTD4, and cytokines including IL-1β, IL-3, tumor necrosis factor (TNF)α, and transforming growth factor(TGF)-β. Emerging evidence indicates a role for IgE-independent MC activation in the late-stage asthmatic response as well as in non-allergic airway diseases including chronic obstructive pulmonary disease (COPD), idiopathic pulmonary fibrosis (IPF), and lung cancer. MC infiltration/activation has been reported in some, but not all, studies of lung cancer. MC-derived TNF-α possesses tumor-suppressive activity while IL-1β supports tumor progression and metastasis. In IPF lungs, an increase in density of tryptase- and chymase-positive MCs (MCTC) and overexpression of TGF-β support the fibrosis progression. MC-derived chymase activates latent TGF-β that induces the differentiation of fibroblasts to matrix-producing myofibroblasts. In summary, increasing evidence highlights a critical role of MCs in non-allergic diseases that may indicate new approaches for therapy.

## Introduction

**Fig. 1:**
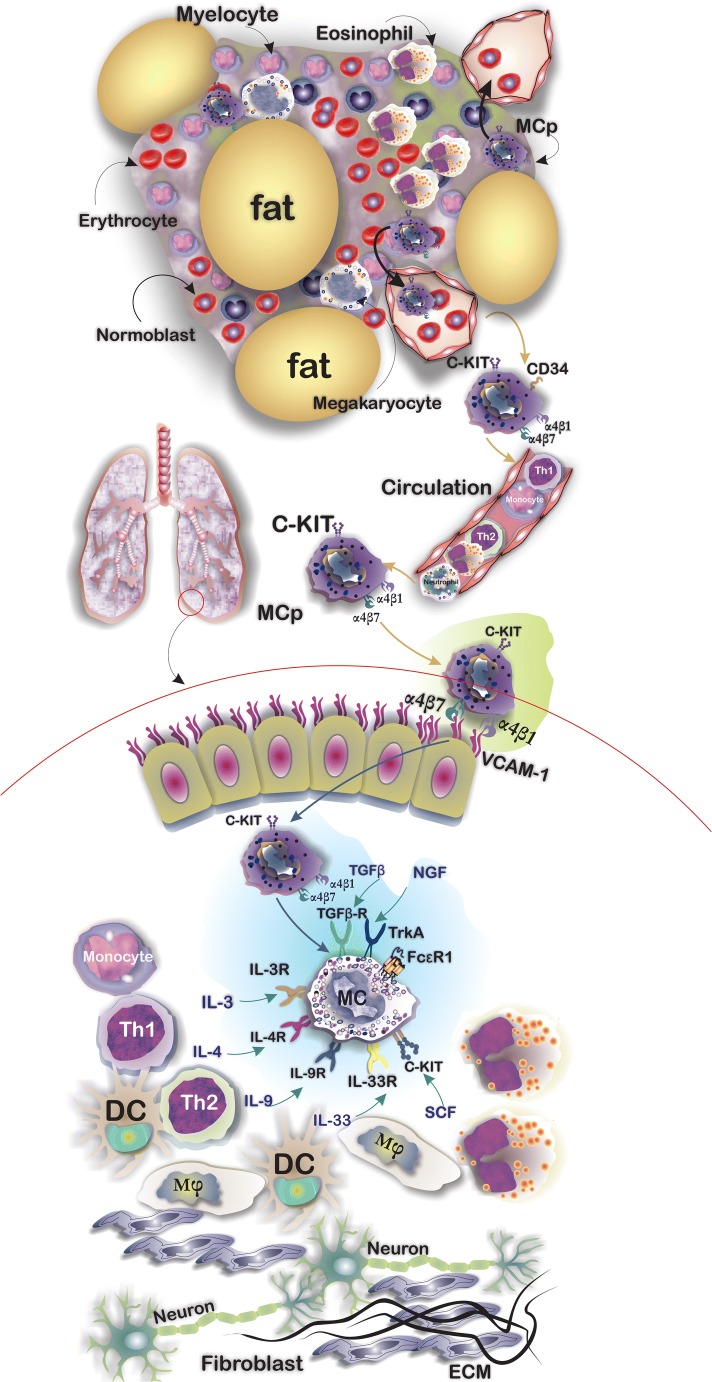
Tissue-resident MCs are the terminally differentiated cells from CD34+/CD117+ MC progenitors (MCps) that originate from the bone marrow. MCps after being released into the circulation reach their target tissues according to a controlled trafficking pattern mostly based on ligand-integrin interactions enter the tissues. MC expression of α4β7 and α4β1 integrins by MCps with vascular cell adhesion molecule (VCAM)-1 expressed by the endothelium of lung tissues, for example, supports their trafficking into the lungs. MCp differentiation to functional and mature MCs depends on several growth factors including stem cell factor (SCF), interleukin (IL)-3, IL-4, IL-9, IL-33, nerve growth factor (NGF), and transforming growth factor (TGF)-β

Mast cells (MCs) are innate immune cells with cytoplasmic granules that are developed from CD34 and CD117 expressing hematopoietic MC progenitor cells (MCps). A well-organized chemokine and integrin-based trafficking system contributes to the migration of MCps from bone marrow where they originate, through the circulation and finally to the target tissues [1]. Expression of integrins, mainly α4β7 and α4β1, on MCps helps them traffic from the circulation into the lung by binding to vascular cell adhesion molecule-1 (VCAM-1) expressed on the endothelium [2]. MCps complete their final stage of differentiation to functional MCs under the influence of growth factors particularly stem cell factor (SCF) [3]. In addition to SCF, there are other cytokines that contribute to the growth and differentiation of MCps to functional mature MCs including transforming growth factor (TGF)β, nerve growth factor (NGF), interleukin (IL)-3, IL-4, IL-9, and IL-33 [2]. Crosslinking of the high-affinity immunoglobulin (Ig)E receptor (FcεRI) by allergen bounded IgE acts as a stimulus for triggers the signaling pathways which result in degranulation of MCs [4] (Fig. 1). Upon degranulation, MCs release a broad spectrum of mediators which are classified in three groups including pre-formed mediators (including histamine, tryptase, and chymase), de novo mediators including prostaglandin (PG)D2, leukotriene (LT)B4, and LTD4, and also a long list of cytokines and growth factors such as tumor necrosis factor (TNF)-α, TGF-β, vascular endothelial growth factor (VEGF), granulocyte-macrophage colony-stimulating factor (GM-CSF), IL-10, IL-8, IL-5, IL-3, and IL-1 [5, 6]. Two major subsets of MCs have been described in humans. These differ according to their secretory granule (SG) protease content and anatomical distribution: tryptase- and chymase-positive MCs (MCTC) which can be mainly found in connective tissues and their SGs contain tryptase, chymase, carboxypeptidase, and cathepsin and a tryptase-positive MC (MCT) subset which are defined by expression of tryptase and absence of chymase and their distribution in the lung and gut [7]. Interestingly, in lung tissues, the MCT subtype releases IL-5 and IL-6, whereas the MCTC subtype expresses IL-4 and IL-13 [8]. Beyond their classic role in IgE-dependent allergic disorders of the lung, MCs may play a role in IgE-independent disorders and contribute to tissue remodeling and reshaping of the lung tumor microenvironment. This review describes MC stimulation and biology and the mechanisms underlying SG release and then describes the evidence for an IgE-independent role of MCs in the chronic lung diseases asthma, chronic obstructive pulmonary disease (COPD), idiopathic pulmonary fibrosis (IPF), and lung cancer.

## Molecular Mechanism of IgE/FcεRI-Mediated Activation of MCs

**Fig. 2:**
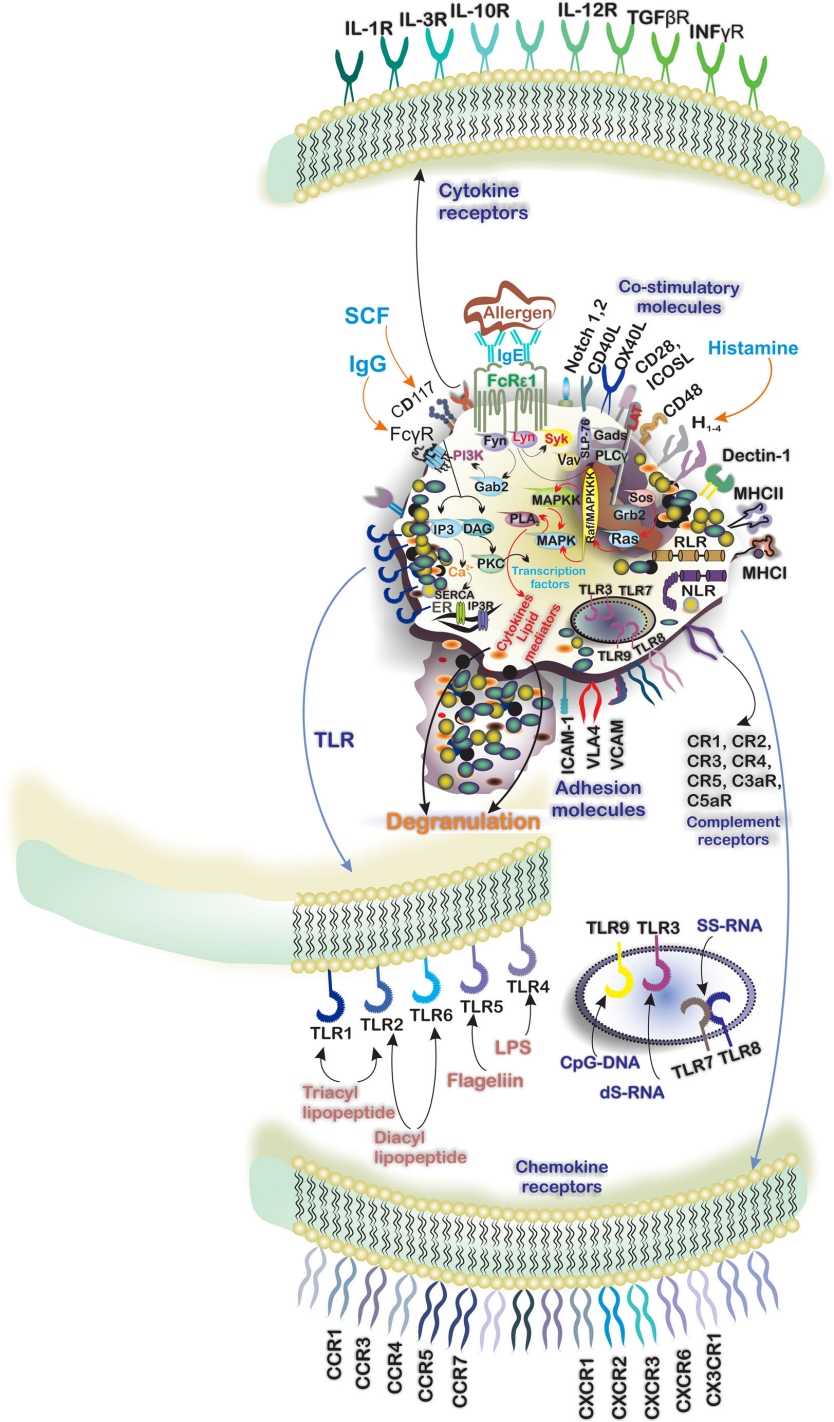
Mast cells (MCs) respond to a wide range of pathologic and environmental stimuli owing to the expression of toll-like receptors (TLRs), NOD-like receptors (NLRs), and RIG-1-like receptors (RLRs). TLR-1, -2, -4, -5, and -6 respond to extracellular ligands mainly cell wall components of bacteria. Moreover, expression of endosomal TLRs including TLR-3, -7, -8, and -9 by MCs enables them to respond to intracellular pathogens including viruses. The expression of receptors for cytokines forms a complicated cytokine network around MCs and plays an important role in MC-immune cells cell-talk. Additionally, MCs express a variety of CCR and CXCR chemokine receptors by which respond to chemokines. IgE/FcεRI signaling has the central role in the degranulation of MCs in response to the allergen. Crosslinking of FcεRI by allergen bounded IgE triggers the signaling pathway and engaging intracellular signaling molecules including Lyn and SYK. Activation of phospholipase Cγ (PLCγ) results in the formation of second messengers including “diacylglycerol” (DAG) and inositol 1,4,5-triphosphate (IP3) and the influx of calcium that induces the degranulation. The consequence of IgE/FcεRI signaling is releasing various mediators including preformed, de novo synthesized, and cytokines

The expression of tetrameric αβγ2 complex of FcεRI by tissue-resident MCs and circulatory basophils enables them to respond to IgE. Physical interaction between IgE and FcεRI is mediated by the α subunit [9]. The β- and γ-chains possess an immunoreceptor tyrosine-based activation motif (ITAM) in their cytoplasmic domains [9] and phosphorylation of these ITAMs by the Src-family protein tyrosine kinase Lyn occurs after IgE/FcεRI engagement. ITAM-bound Syk and Fyn can enhance Lyn-mediated phosphorylation and indeed several protein tyrosine kinases (PTKs) promote phosphorylation and activation of downstream signaling molecules including transmembrane adaptor proteins (TRAPs) and linker for activation of T cells (LAT). Phosphorylated TRAPs act as membrane docking points for SH2 domains containing proteins such as Grb2 and the subsequent recruitment and activation of phospholipase Cγ (PLCγ). PLCγ produces several second messengers including diacylglycerol” (DAG) and inositol 1,4,5-triphosphate (IP3) following modulation of phosphatidylinositol 4,5-biphosphate” (PIP2). IP3, which induces Ca2+ efflux from the endoplasmic reticulum, together with the calcium sensor stromal interaction molecule 1 (STIM1)/ORAI1, results in enhanced intracellular Ca2+ levels, MC degranulation, and the release of a wide spectrum of mediators into the surrounding microenvironment [10–13] (Fig. 2). The importance of IgE/FcεRI signaling in the activation and degranulation of MCs has led to several strategies for the pharmaceutical control of MC activation which are summarized in Table 1.

**Table 1:** Strategies introduced to interfere IgE/FcεRI signaling

Strategy	Pharmaceutical agents/Abs	Description/mechanism of action	Ref
Anti-IgE monoclonal antibodies	Omalizumab	Recombinant humanized anti-IgE mAb, also known as Xolair® Approved by FDA for the treatment of mild allergic asthma Mechanism of action: binds to free IgE and decreases the availability of IgE available to interact with FcεRI Omalizumab has no interference with receptor-bound IgE	[57, 58]
	Ligelizumab (QGE031)	Humanized IgG1 mAb with ability to binding to the Cε3 domain of IgE	[59]
	Quilizumab	Structurally is a humanized, monoclonal IgG1 antibody with the ability to bind membrane-bound but not soluble IgE due to the absence of M1-prime segment in soluble IgE	[60]
Designed ankyrin repeat proteins (DARPins)	DARPin E2_79	Acts by preventing IgE-FcεRI binding and disrupting preformed IgE-FcεRI complexes in vitro	[57]
	DARPin E3_54	Inhibits IgE-FcεRI interaction	[57]
	DARPin 30/85	Bispecific molecule capable of binding to FcεRIα	[57]
Co-aggregation of FcεRI with the inhibitory FcγRIIb	GE2	GE2 structurally is comprised of the hinge-Cγ2-Cγ3 domains from IgG Fc linked to Cε2-Cε4 domains of human IgE Fc Inhibits the phosphorylation of Syk	[57]

## MC Expression and Degranulation by TLRs and Lectin Receptors

**Fig. 3:**
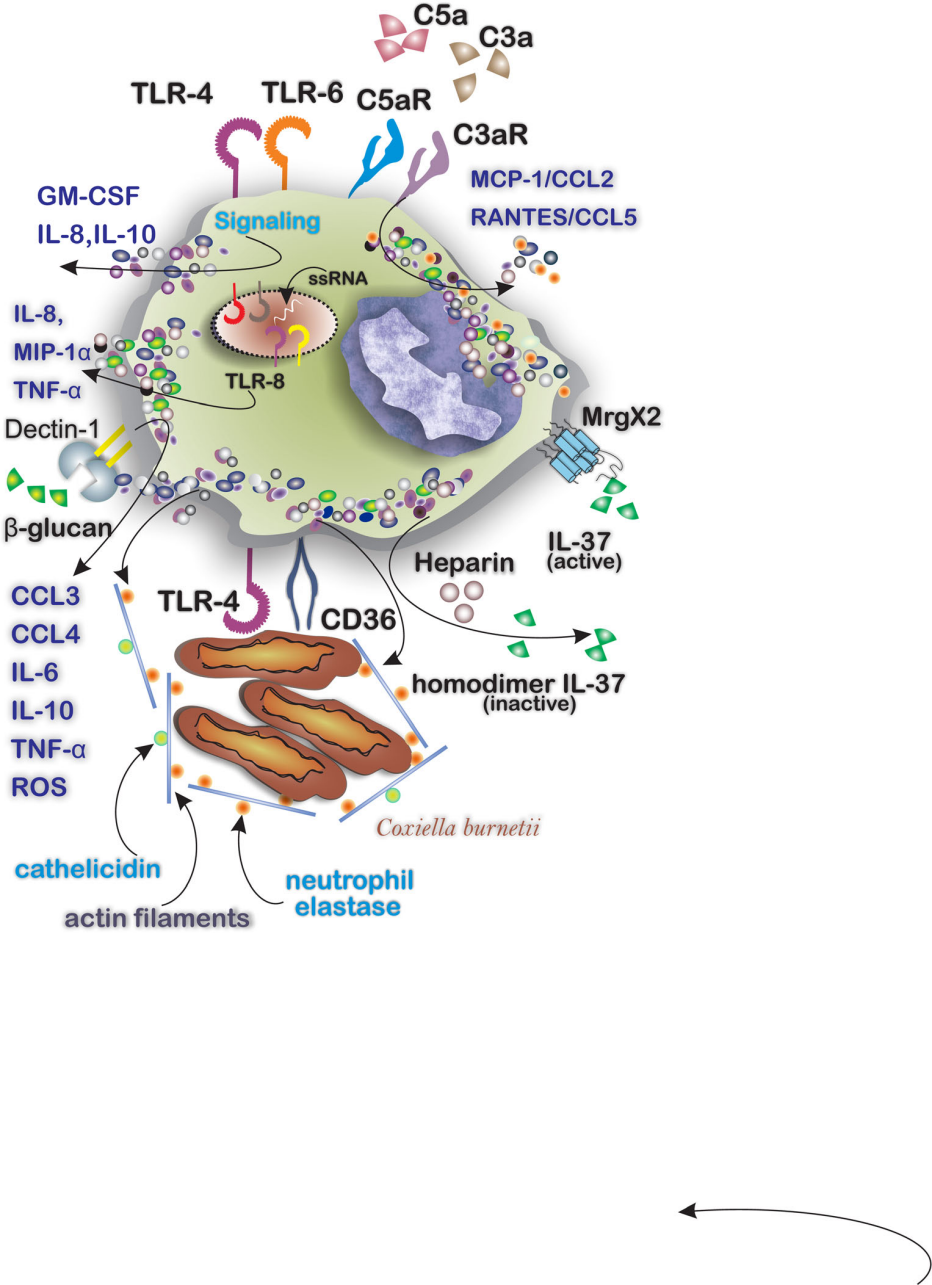
Cytokines, exogenous chemical components, and a variety of microbes can activate MCs. The mediator releasing pattern depends on engaged receptors including TLRs, complement receptors, and cytokine receptors. MCs respond to a variety of microbes by expressing surface receptors to recognize the pathogens, i.e., MCs respond to the presence of *Coxiella burnetii* by CD36 and TLR4 and release neutrophil elastase/cathelicidin attached to actin filaments around the *Coxiella burnetii* to confine the infection

Although IgE/FcεRI signaling is the main pathway through which MCs become activated and degranulate MCs express other receptors that can induce cell activation [14], these receptors make MCs highly effective responders to different endogenous and exogenous airborne factors. Early mRNA studies showed that human and mice MCs express several toll-like receptors (TLRs) including TLR1, TLR-2, TLR-4, and TLR-6 through which they respond to lipopolysaccharide (LPS) and peptidoglycan to produce LTC4, TNF-α, IL-1β, IL5, IL-13, and GM-CSF [15]. Indeed, the expression of TLRs enables MCs to act as sentinel and responding cells when they encounter environmental pathogens. Activation of TLR-4 and TLR-6 on MCs results in the upregulation of cytokines mainly GM-CSF, IL-8, and IL-10. Additionally, the engagement of TLR-8 by single strand-RNA induces the release of IL-8, MIP-1α, and TNF-α [16]. MCs also express C-type lectin receptors including the type II transmembrane β-glucan receptor Dectin-1 which is involved in microbial defense. Exposure of MCs to yeast *C. albicans* results in MC degranulation and the release of reactive oxygen species (ROS) and cytokines including CCL3, CCL4, IL-6, IL-10, and TNF-α. Blockade of Dectin-1, but not of TLR-2, confirmed the key role of Dectin-1 in the release of TNFα in response to *C. albicans* [17]. Engagement and activation of MC-expressed scavenger receptor CD36 and of TLR-4 are implicated in the microbial defense against *Coxiella burnetii* by MCs. This leads to the release of actin filaments (cytonemes) bound to cathelicidin and of neutrophil elastase which enables the capture and elimination of *Coxiella burnetii* [18] (Fig. 3).

## MC Activation by Complement Receptors and Other GPCRs

MCs express several complement receptors and receptors to anaphylatoxins including C3aR and C5aR (CD88) [19, 20]. Activation of MCs with C3a, for example, induces the release of MCP-1/CCL2 and RANTES/CCL5 in LAD2 cells [20]. IL-37 possesses antimicrobial properties and promotes physiologic processes including inflammation and angiogenesis. IL-37-mediated MC activation occurs through the G protein-coupled receptor MrgX2 in human MCs and is considered a promising new treatment in MC-driven disorders [21]. Interestingly, MC-derived heparin may inactivate IL-37 by homodimerization with macrophage-derived IL-37 in response to TLR activation [22, 23]. Corticotropin-releasing factor receptor subtype 1 (CRFR1) expressed on MCs interacts with its ligand corticotropin-releasing factor (CRF) to enhance MC degranulation [24]. Endogenous factors include several cytokines and neuropeptides which stimulate MC degranulation when they engage with the corresponding receptors on MCs (Table 2). The proximity of nerves and MCs within the perivascular space in organs such as the heart may lead to activation of MCs by factors such as substance P released by neurons. Levick et al. reported that substance P activates cardiac MCs via cell surface neurokinin-1 receptors to induce the release of histamine [25]. Similarly, mucosal MCs and nerve fibers containing calcitonin gene-related peptide (CGRP) are in close proximity within the colon of mice with food allergy (FA). Kim and colleagues studied the effects of CGRP on MCs using a CGRP-receptor antagonist, BIBN4096BS and concluded that blockade of the CGRP/CGRP receptor (CGRPR) interaction alleviates allergic symptoms [26]. Yang et al. described a mastocytosis-like disease in mice following MC stimulation by NGF and subsequent binding to its receptor tropomyosin receptor kinase A (TrkA) [27].

**Table 2:** Cytokines and neuropeptides that are capable of inducing MC degranulation

Mediator	Receptor, interaction, and results	Ref
SCF	Binds to c-KIT (CD117), induces MC differentiation, survival, and release of pro-inflammatory cytokines, mainly IL-6, IL-8, and TNF-α	[61, 62]
IL-33	Acts through ST2 and induces the release of CCL4 and CXCL8	[63]
Substance P	Acts through NK1R and induces the release of IL-17 and histamine	[25, 64]
Nerve growth factor (NGF)	Binds to TrkA, induces MC hyperplasia and cytokine production Induces the release of β-hexosaminidase in mice peritoneal MC	[65] [66]
Calcitonin gene-related peptide (CGRP)	Acts through CGRPR and induces serotonin release	[67]

## Control of Secretory Granule Release by MC Receptors

As described above, human MCs are heterogeneous and are often characterized by the neutral protease content within granules, e.g., MCTC skin mast cells and MCT lung mast cells [28]. MC granule subpopulations can be further defined at the ultrastructural and protein content level. Thus, type I granules contain MHC class II, β-hexosaminidase, Lysosomal-associated membrane protein (LAMP)-1 and -2, and the mannose 6 phosphate receptor (M6PR); type II granules contain these mediators plus serotonin and type III granules contain β-hexosaminidase and serotonin but not MHC class II [29]. The triggering of SG release by various mast cell stimuli is complex and tightly regulated [30]. In its most simplistic form, the fusion of the SG membrane with the cell plasma membrane can allow SG contents to be released into the extracellular space. Besides, three other distinct types of SG degranulation have been described: (i) “Kiss-and-run” exocytosis allows partial release of the SG cargo through the formation of a transient fusion pore; (ii) “Piecemeal exocytosis” using vesicles to shuttle SG stored mediators to the plasma membrane; and (iii) “compound exocytosis” or “anaphylactic degranulation” wherein fused SG-SG multi-granular structures merge with the plasma membrane [30]. These distinct types of SG content release can elicit the rapid and complete release of contents (compound exocytosis) while piecemeal degranulation allows the precise release of specific SG components. Different MC triggers or stimuli allow these distinct SG release processes to occur. For example, the degranulation responses triggered by Mas-related gene x2 (MRGX2) and FceRI differ in the quality and degree of mediator release [31] with reduced cytokine (TNF, IL-13, VEGF, and MCP-1) and eicosanoid (PGE2 and PGD2) release being less following MRGX2 stimulation despite comparable levels of MC degranulation. Thus, the specificity and extent of MC degranulation are stimulus-dependent and regulated by the plethora of MC cell surface receptors. Mechanistically, MC SG exocytosis requires SNAREs [soluble NSF (N-ethylmaleimide-sensitive factor) attachment protein receptors] and SM (Sec1-Munc18) proteins [29]. Multiple sets of SNARE-SM complexes exist which differentially regulate both constitutive and regulated SG membrane fusion and mediator release. Support for stimulus/SNARE specificity derives from the inhibition of specific SNARE proteins selectivity suppressed the release of CXCL8, CCL2, and CCL3 from IgE/FcεRI-stimulated MCs [32]. This suggests that different MC stimuli may exploit distinct exocytic fusion machinery to induce the selective release of mediators from SGs although this needs to be confirmed [29].

## The Role of MCs in Non-allergic Airway Disease

### Role of MCs in Non-allergic Asthma

MCs are the main innate immune cells orchestrating allergic asthma when allergen-specific IgE sensitizes MCs by binding to FcεRI. Further exposure to the same allergen evokes MC degranulation of MCs. However, IgE-independent interactions at both cellular and molecular levels can affect the progression and maintenance of inflammation and allergy in the lungs. MC activation during allergic asthma may also result from factors released by other cell types in an IgE-independent manner. In this regard, allergen exposure induces the release of IL-25, IL-33, and thymic stromal lymphopoietin (TSLP) from the airway epithelium. IL-33, acting through ST2 on the MC, triggers the MyD88 signaling pathway resulting in the activation of the mitogen-activated protein (MAP) kinase and nuclear factor kappa-light-chain-enhancer of activated B cells (NF-κB) pathways. These pathways consequently result in MC activation, proliferation, and the release of LTs [33]. In chronic asthma, MCs may contribute to airway remodeling through the release of factors including tryptase which induces the production of type I collagen by fibroblasts. Moreover, tryptase and histamine together promote the proliferation of airway smooth muscle (ASM) cells [34]. Interestingly, tryptase released from MCs may also activate the pro-metalloproteinase (MMP)-1 produced by ASM to form active MMP1 [35]. The levels of MMP1 in individuals with asthma correlate with bronchial hyperresponsiveness (BHR) and exacerbation of disease [35].

### Role of MCs in the Pathology of COPD

The umbrella term Chronic Obstructive Pulmonary Disease (COPD) is used to describe a set of symptoms mainly chronic bronchitis and airway obstruction, remodeling of the small airways, and emphysema, which results in an irreversible decline in the function of the pulmonary system [36]. Infiltration of a variety of inflammatory cell types including neutrophils and macrophages into the small airways is a common finding in COPD [36]. Ekberg-Jansson et al. investigated the distribution of MCs in different bronchial compartments of 29 asymptomatic smokers and 16 healthy non-smokers. The smoking group had higher numbers of infiltrating MCs in the epithelium, lamina propria, and smooth muscle compared with the non-smoking group. Besides, the intensity of structural and histologic changes such as epithelial integrity and thickness of the laminin layer was correlated with MC density [37]. Furthermore, individuals with COPD have a lower density of MCT in the subepithelial area of the central airways comparing with healthy controls and the density of MCT correlates positively with the forced expiratory volume in 1 s (FEV1)/forced vital capacity (FVC) ratio [38]. In contrast, Kosanovic and colleagues reported that chymase-positive MCs increased particularly in the perivascular regions of the lungs and that their number was positively correlated with better lung function in individuals with COPD [39]. In a rat model of COPD induced by nitrogen dioxide inhalation, inhibition of MCs by sodium cromoglycate prevented nerve-mediated bronchial smooth muscle hyperactivity [40]. Mechanistically, this required the A2B subtype of adenosine receptor on MCs, which induced histamine release and smooth muscle contraction [40]. Finally, MCs may be involved in the functional effects of the enhanced IL-17A expression reported in COPD. IL-17A, acting through IL-17 receptors (IL-17RA and IL-17RC) on MCs, induces the release of the proangiogenic factors fibroblast growth factor (FGF)-2 and VEGF to mediate vascular remodeling [41].

### Distribution of MCs and their Function in Idiopathic Pulmonary Fibrosis

Idiopathic pulmonary fibrosis (IPF) is a chronic progressive fibrotic lung disease with a poorly understood etiology [42]. Induction of myofibroblast apoptosis usually occurs following the completion of scarring and contributes to the control of the degree of fibrosis. However, myofibroblasts continue to produce excessive amounts of matrix components during aberrant wound healing processes [43]. The number of infiltrating MCs is increased in IPF lungs than in normal healthy lungs and infiltrating MC numbers correlates with the degree of fibrosis [44]. Mechanistically, this may involve MC-derived chymase which can induce fibroblast proliferation [43, 45]. In fibrotic lung diseases, the interplay between MCs and fibroblasts contributes to the formation of a profibrotic milieu in which fibroblasts support MC survival and proliferation by producing SCF and in turn, MC-derived chymase activates latent TGF-β1 which mediates fibroblast to myofibroblast differentiation [46, 47]. The distribution of MC subsets has been investigated and the MCT population remains unchanged while the MCTC population is increased in healthy parenchyma of IPF patients. In contrast, both subtypes were increased significantly in fibrotic parenchyma [47].

### Role of MCs in Lung Cancer

MC accumulation within the tumor microenvironment (TME) is mediated by SCF and CCL15 released from tumor cells [48]. MCs may also contribute to shaping the TME by releasing a variety of angiogenic mediators including chymase, tryptase, VEGF, IL-6, FGF-2, and platelet-derived growth factor (PDGF). MC release of MMP9 can induce ECM degradation and thereby support tumor progression and metastasis [48]. In non-small cell lung cancer (NSCLC), there is a greater infiltration of both MCT and MCTC in patients with extended survival comparing with patients with poor survival. Both MC subtypes produce TNF-α which plays a role in anti-tumor immune responses [8]. Qu and colleagues demonstrated that human MC migration in vitro was enhanced by the release of CCL5 from the NSCLC cell lines A549 and SPC-α-1. Furthermore, in vivo transplantation of a human MC cell line (HMC-1) together with A549 cells into nude mice showed that MCs support tumor progression through their release of IL-8 which induced β-catenin phosphorylation in NSCLC cells [49]. MCs also enhanced epithelial-to-mesenchymal transition which is important in tumor migration and progression [49]. Infiltration of MCs has also been reported in human lung adenocarcinoma (LADC) tissues. Lilis and co-workers applied three KRAS-mutant LADC models to two MC-deficient mouse strains (cKitWsh and Cpa3.Cre) [50]. They showed that MCs infiltrate into tumors and that a MC deficiency protects the mice from tumor initiation within the airway and alveolar epithelium following induction with the carcinogen urethane or the oncogenic KRASG12D mutant respectively. Enhanced tumor progression required IL-1β release from KIT+ MCs localized within the TME [50].

## MMP9, MCs, and Airway Remodeling in Airway Disease

It is unknown whether the MC release of MMP9 in cancer is mimicked in other diseases. However, there is some evidence that MMPs may play a role in MC-regulated remodeling. In a mouse model of asthma, *Clostridium butyricum* CGMCC0313-1 (*C. butyricum*) significantly reduced lung function, airway inflammation, mast cell degranulation, and airway remodeling. These effects were associated with reduced MMP9 expression [51]. Besides, there is a strong correlation between MMP9 expression and mast cell numbers after nasal allergen challenge in subjects with allergic rhinitis [52]. Furthermore, the IL-3-stimulated release of MM9 by murine MCs is essential for MC migration into tissues and suppressed by stem cell factor [53]. This suggests that there is an interplay between stem cell factor, MMP9, and mast cell engagement with tissue matrix. Other MMPs such as MMP10 have been associated with MC activation, airway remodeling, and extracellular matrix (ECM) organization in severe asthma [54], while airway smooth muscle-derived MMP1 was activated by MC tryptase enabling the development of a proliferative ECM that may account for the association with a link to airway responsiveness in severe asthma [35]. Finally, activated MCs in 3D culture resulted in enhanced release of active MMP2 and ASM proliferation [55] and of collagen and MMP2 and MMP3 release and fibroblast contraction [56].

## Conclusion

Beyond their well-documented role in allergy, MCs are also involved in the pathology of non-allergic diseases including autoimmune disorders, obesity, infertility, and even physiologic processes such as wound healing. They owe much of this ability to the expression of a wide spectrum of cell surface receptors including those for complement and various cytokines. MCs release a plethora of mediators in a stimulus-dependent manner despite similar degrees of degranulation. This enables them to crosstalk with other immune/non-immune cells. Studying the role of MCs in pathology of non-allergic diseases is not simple due to different anatomic distribution of MCs and MC subtypes, different immune responses to different stimuli, their ability to release both pro-inflammatory and anti-inflammatory cytokines in a context-dependent manner, limited number of MC-deficient mice models for each disease, inconsistent results of in vivo and in vitro investigations, and the limited number of mice and human cell lines. However, careful design of experiments and future single-cell RNA-sequence analysis of individual MCs from patient samples at baseline and in response to therapies will help define MC involvement in disease. New in vitro and in vivo models that utilize this information can then be derived to provide further insight into this important area.

## Abbreviations

ASM: Airway smooth muscle
BAL: Bronchoalveolar lavage
BHT: Butylated hydroxytoluene
CGRP: Calcitonin gene related peptide
COPD: Chronic obstructive pulmonary disease
CRF: Corticotropin-releasing factor (CRF)
CRFR1: Corticotropin-releasing factor receptor subtype 1
DAG: Diacylglycerol
FEV1: Forced expiratory volume in 1 s
FGF: Fibroblast growth factor
FGF-2: Fibroblast growth factor 2
Foxf1: Forkhead Box protein F1
FVC: Forced vital capacity
GM-CSF: Granulocyte-macrophage colony-stimulating factor
IP3: Inositol 1,4,5-triphosphate
IPF: Idiopathic pulmonary fibrosis
ITAM: Immunoreceptor tyrosine-based activation motif
LAT: Linker for activation of T cells
LT: Leukotriene
MCL: Mast cell leukemia
MCp: Mast cell progenitor
MIP-1α: Macrophage inflammatory protein 1α
NGF: Nerve growth factor
NLR: Nod-like receptors
PDGF: Platelet-derived growth factor
PG: Prostaglandin
PTKs: Protein tyrosine kinases
RANTES: Regulated upon activation, normal T cell expressed, and secreted
RIG-I: Retinoic acid-inducible gene-I
RLR: RIG-I-like Receptor
ROS: Reactive oxygen species
SCF: Stem cell factor
SM: Systemic mastocytosis
STIM1: Calcium sensor stromal interaction molecule 1
TGFβ: Transforming growth factor-β
TNF-α: Tumor necrosis factor-α
TRAPs: Transmembrane adaptor proteins
Trka: Tropomyosin receptor kinase
TSLP: Thymic stromal lymphopoietin
VEGF: Vascular endothelial growth factor

## References

[1] Mortaz E, Amani S, Mumby S, Adcock IM, Movassaghi M, Folkerts J, Garssen J, Folkerts G (2018). Role of mast cells and type 2 innate lymphoid (ILC2) cells in lung transplantation. J Immunol Res.

[2] Elieh Ali Komi D, Ribatti D (2019). Mast cell-mediated mechanistic pathways in organ transplantation. Eur J Pharmacol.

[3] Redegeld FA, Yu Y, Kumari S, Charles N, Blank U (2018). Non-IgE mediated mast cell activation. Immunol Rev.

[4] Yu Y, Blokhuis BR, Garssen J, Redegeld FA (2016). Non-IgE mediated mast cell activation. Eur J Pharmacol.

[5] Yu Yingxin, Blokhuis Bart, Garssen Johan, Redegeld Frank (2019). A Transcriptomic Insight into the Impact of Colon Cancer Cells on Mast Cells. International Journal of Molecular Sciences.

[6] Elieh Ali Komi D, Sharma L, Dela Cruz CS (2018). Chitin and its effects on inflammatory and immune responses. Clin Rev Allergy Immunol.

[7] Elieh Ali Komi D, Bjermer L (2019). Mast cell-mediated orchestration of the immune responses in human allergic asthma: current insights. Clin Rev Allergy Immunol.

[8] Shikotra A, Ohri C, Green R, Waller D, Bradding P (2016). Mast cell phenotype, TNFα expression and degranulation status in non-small cell lung cancer. Sci Rep.

[9] Sutton BJ, Davies AM (2015). Structure and dynamics of IgE-receptor interactions: FcepsilonRI and CD23/FcepsilonRII. Immunol Rev.

[10] Blank U, Rivera J (2004). The ins and outs of IgE-dependent mast-cell exocytosis. Trends Immunol.

[11] Draber P, Sulimenko V, Draberova E (2012). Cytoskeleton in mast cell signaling. Front Immunol.

[12] Siraganian RP, de Castro RO, Barbu EA, Zhang J (2010). Mast cell signaling: the role of protein tyrosine kinase Syk, its activation and screening methods for new pathway participants. FEBS Lett.

[13] Potuckova L, Draberova L, Halova I, Paulenda T, Draber P (2018). Positive and negative regulatory roles of C-terminal Src kinase (CSK) in FcepsilonRI-mediated mast cell activation, independent of the transmembrane adaptor PAG/CSK-binding protein. Front Immunol.

[14] Elieh-Ali-Komi D, Cao Y (2017). Role of mast cells in the pathogenesis of multiple sclerosis and experimental autoimmune encephalomyelitis. Clin Rev Allergy Immunol.

[15] Kulka M, Alexopoulou L, Flavell RA, Metcalfe DD (2004). Activation of mast cells by double-stranded RNA: evidence for activation through toll-like receptor 3. J Allergy Clin Immunol.

[16] Suurmond J, Dorjee AL, Knol EF, Huizinga TW, Toes RE (2015). Differential TLR-induced cytokine production by human mast cells is amplified by FcvarepsilonRI triggering. Clin Exp Allergy.

[17] Nieto-Patlan A, Campillo-Navarro M, Rodriguez-Cortes O, Munoz-Cruz S, Wong-Baeza I, Estrada-Parra S, Estrada-Garcia I, Serafin-Lopez J, Chacon-Salinas R (2015). Recognition of Candida albicans by Dectin-1 induces mast cell activation. Immunobiology.

[18] Mezouar S, Vitte J, Gorvel L, Ben Amara A, Desnues B, Mege JL (2019) Mast cell cytonemes as a defense mechanism against Coxiella burnetii. mBio 10(2). 10.1128/mBio.02669-18 10.1128/mBio.02669-18 PMC6469977 30992359

[19] de Vries MR, Wezel A, Schepers A, van Santbrink PJ, Woodruff TM, Niessen HW, Hamming JF, Kuiper J, Bot I, Quax PH (2013). Complement factor C5a as mast cell activator mediates vascular remodelling in vein graft disease. Cardiovasc Res.

[20] Venkatesha RT, Berla Thangam E, Zaidi AK, Ali H (2005). Distinct regulation of C3a-induced MCP-1/CCL2 and RANTES/CCL5 production in human mast cells by extracellular signal regulated kinase and PI3 kinase. Mol Immunol.

[21] Subramanian H, Gupta K, Guo Q, Price R, Ali H (2011). Mas-related gene X2 (MrgX2) is a novel G protein-coupled receptor for the antimicrobial peptide LL-37 in human mast cells: resistance to receptor phosphorylation, desensitization, and internalization. J Biol Chem.

[22] Eisenmesser EZ, Gottschlich A, Redzic JS, Paukovich N, Nix JC, Azam T, Zhang L, Zhao R, Kieft JS, Meng X, Dinarello CA, The E (2019). Interleukin-37 monomer is the active form for reducing innate immunity. Proc Natl Acad Sci U S A.

[23] Theoharides Theoharis C., Tsilioni Irene, Conti Pio (2019). Mast Cells May Regulate The Anti-Inflammatory Activity of IL-37. International Journal of Molecular Sciences.

[24] Ayyadurai S, Gibson AJ, D’Costa S, Overman EL, Sommerville LJ, Poopal AC, Mackey E, Li Y, Moeser AJ (2017). Frontline science: corticotropin-releasing factor receptor subtype 1 is a critical modulator of mast cell degranulation and stress-induced pathophysiology. J Leukoc Biol.

[25] Levick SP, Brower GL, Janicki JS (2019). Substance P-mediated cardiac mast cell activation: an in vitro study. Neuropeptides.

[26] Kim JH, Yamamoto T, Lee J, Yashiro T, Hamada T, Hayashi S, Kadowaki M (2014). CGRP, a neurotransmitter of enteric sensory neurons, contributes to the development of food allergy due to the augmentation of microtubule reorganization in mucosal mast cells. Biomed Res (Tokyo, Japan).

[27] Yang M, Pan Z, Huang K, Busche G, Feuerhake F, Chaturvedi A, Nie D, Heuser M, Thol F, von Neuhoff N, Ganser A, Li Z (2017). Activation of TRKA receptor elicits mastocytosis in mice and is involved in the development of resistance to KIT-targeted therapy. Oncotarget.

[28] Olivera A, Beaven MA, Metcalfe DD (2018). Mast cells signal their importance in health and disease. J Allergy Clin Immunol.

[29] Xu H, Bin N-R, Sugita S (2018). Diverse exocytic pathways for mast cell mediators. Biochem Soc Trans.

[30] Espinosa E, Valitutti S (2018). New roles and controls of mast cells. Curr Opin Immunol.

[31] Gaudenzio N, Sibilano R, Marichal T, Starkl P, Reber LL, Cenac N, McNeil BD, Dong X, Hernandez JD, Sagi-Eisenberg R (2016). Different activation signals induce distinct mast cell degranulation strategies. J Clin Invest.

[32] Frank SP, Thon K-P, Bischoff SC, Lorentz A (2011). SNAP-23 and syntaxin-3 are required for chemokine release by mature human mast cells. Mol Immunol.

[33] Saluja R, Khan M, Church MK, Maurer M (2015). The role of IL-33 and mast cells in allergy and inflammation. Clin Transl Allergy.

[34] Modena BD, Dazy K, White AA (2016). Emerging concepts: mast cell involvement in allergic diseases. Transl Res.

[35] Naveed SU, Clements D, Jackson DJ, Philp C, Billington CK, Soomro I, Reynolds C, Harrison TW, Johnston SL, Shaw DE, Johnson SR (2017). Matrix metalloproteinase-1 activation contributes to airway smooth muscle growth and asthma severity. Am J Respir Crit Care Med.

[36] Wang Y, Xu J, Meng Y, Adcock IM, Yao X (2018). Role of inflammatory cells in airway remodeling in COPD. Int J Chron Obstruct Pulmon Dis.

[37] Ekberg-Jansson A, Amin K, Bake B, Rosengren A, Tylen U, Venge P, Lofdahl CG (2005). Bronchial mucosal mast cells in asymptomatic smokers relation to structure, lung function and emphysema. Respir Med.

[38] Gosman MM, Postma DS, Vonk JM, Rutgers B, Lodewijk M, Smith M, Luinge MA, Ten Hacken NH, Timens W (2008). Association of mast cells with lung function in chronic obstructive pulmonary disease. Respir Res.

[39] Kosanovic D, Dahal BK, Peters DM, Seimetz M, Wygrecka M, Hoffmann K, Antel J, Reiss I, Ghofrani HA, Weissmann N, Grimminger F, Seeger W, Schermuly RT (2014). Histological characterization of mast cell chymase in patients with pulmonary hypertension and chronic obstructive pulmonary disease. Pulm Circ.

[40] Kuzubova NA, Lebedeva ES, Titova ON, Fedin AN, Dvorakovskaya IV (2017). Role of mast cells in bronchial contraction in nonallergic obstructive lung pathology. J. Smooth Muscle Res..

[41] Roos AB, Mori M, Gura HK, Lorentz A, Bjermer L, Hoffmann HJ, Erjefalt JS, Stampfli MR (2017). Increased IL-17RA and IL-17RC in end-stage COPD and the contribution to mast cell secretion of FGF-2 and VEGF. Respir Res.

[42] Pan RL, Swigris JJ, Zhao YW, Guo AM, Wu Q, Li SJ (2019). Reliability and validity of Chinese version of a tool to assess the quality of life in idiopathic pulmonary fibrosis in patients with interstitial lung disease. Int J Nurs Sci.

[43] Komi DEA, Khomtchouk K, Santa Maria PL (2019) A review of the contribution of mast cells in wound healing: involved molecular and cellular mechanisms. Clin Rev Allergy Immunol:1–15. 10.1007/s12016-019-08729-w 10.1007/s12016-019-08729-w 30729428

[44] Cha SI, Chang CS, Kim EK, Lee JW, Matthay MA, Golden JA, Elicker BM, Jones K, Collard HR, Wolters PJ (2012). Lung mast cell density defines a subpopulation of patients with idiopathic pulmonary fibrosis. Histopathology.

[45] Kosanovic D, Luitel H, Dahal BK, Cornitescu T, Janssen W, Danser AH, Garrelds IM, De Mey JG, Fazzi G, Schiffers P, Iglarz M, Fischli W, Ghofrani HA, Weissmann N, Grimminger F, Seeger W, Reiss I, Schermuly RT (2015). Chymase: a multifunctional player in pulmonary hypertension associated with lung fibrosis. Eur Respir J.

[46] Shimbori C, Upagupta C, Bellaye PS, Ayaub EA, Sato S, Yanagihara T, Zhou Q, Ognjanovic A, Ask K, Gauldie J, Forsythe P, Kolb MRJ (2019). Mechanical stress-induced mast cell degranulation activates TGF-beta1 signalling pathway in pulmonary fibrosis. Thorax.

[47] Andersson CK, Andersson-Sjoland A, Mori M, Hallgren O, Pardo A, Eriksson L, Bjermer L, Lofdahl CG, Selman M, Westergren-Thorsson G, Erjefalt JS (2011). Activated MCTC mast cells infiltrate diseased lung areas in cystic fibrosis and idiopathic pulmonary fibrosis. Respir Res.

[48] Komi DEA, Redegeld FA (2019) Role of mast cells in shaping the tumor microenvironment. Clin Rev Allergy Immunol:1–13 10.1007/s12016-019-08753-w PMC7244463 31256327

[49] Qu J, Cheng T, Liu L, Heng J, Liu X, Sun Z, Wang W, Li K, Yang N (2019). Mast cells induce epithelial-to-mesenchymal transition and migration in non-small cell lung cancer through IL-8/Wnt/beta-catenin pathway. J Cancer.

[50] Lilis I, Ntaliarda G, Papaleonidopoulos V, Giotopoulou GA, Oplopoiou M, Marazioti A, Spella M, Marwitz S, Goldmann T, Bravou V, Giopanou I, Stathopoulos GT (2019). Interleukin-1beta provided by KIT-competent mast cells is required for KRAS-mutant lung adenocarcinoma. Oncoimmunology.

[51] Juan Z, Zhao-Ling S, Ming-Hua Z, Chun W, Hai-Xia W, Meng-Yun L, Jian-Qiong H, Yue-Jie Z, Xin S (2017). Oral administration of Clostridium butyricum CGMCC0313-1 reduces ovalbumin-induced allergic airway inflammation in mice. Respirology.

[52] Mori S, Pawankar R, Ozu C, Nonaka M, Yagi T, Okubo K (2012). Expression and roles of MMP-2, MMP-9, MMP-13, TIMP-1, and TIMP-2 in allergic nasal mucosa. Allergy, Asthma Immunol Res.

[53] Tanaka A, Arai K, Kitamura Y, Matsuda H (1999). Matrix metalloproteinase-9 production, a newly identified function of mast cell progenitors, is downregulated by c-kit receptor activation. Blood.

[54] Kuo Chih-Hsi S., Pavlidis Stelios, Zhu Jie, Loza Matthew, Baribaud Fred, Rowe Anthony, Pandis Ioannis, Gibeon David, Hoda Uruj, Sousa Ana, Wilson Susan J., Howarth Peter, Shaw Dominick, Fowler Stephen, Dahlen Barbro, Chanez Pascal, Krug Norbert, Sandstrom Thomas, Fleming Louise, Corfield Julie, Auffray Charles, Djukanovic Ratko, Sterk Peter J., Guo Yike, Adcock Ian M., Chung Kian Fan (2019). Contribution of airway eosinophils in airway wall remodeling in asthma: Role ofMMP-10andMET. Allergy.

[55] Ceresa CC, Knox AJ, Johnson SR (2009). Use of a three-dimensional cell culture model to study airway smooth muscle-mast cell interactions in airway remodeling. Am J Phys Lung Cell Mol Phys.

[56] Margulis A, Nocka KH, Wood NL, Wolf SF, Goldman SJ, Kasaian MT (2009). MMP dependence of fibroblast contraction and collagen production induced by human mast cell activation in a three-dimensional collagen lattice. Am J Phys Lung Cell Mol Phys.

[57] Gomez G (2019). Current strategies to inhibit high affinity FcepsilonRI-mediated signaling for the treatment of allergic disease. Front Immunol.

[58] Eggel A, Baravalle G, Hobi G, Kim B, Buschor P, Forrer P, Shin JS, Vogel M, Stadler BM, Dahinden CA, Jardetzky TS (2014). Accelerated dissociation of IgE-FcepsilonRI complexes by disruptive inhibitors actively desensitizes allergic effector cells. J Allergy Clin Immunol.

[59] Kocaturk E, Zuberbier T (2018). New biologics in the treatment of urticaria. Curr Opin Allergy Clin Immunol.

[60] Harris JM, Maciuca R, Bradley MS, Cabanski CR, Scheerens H, Lim J, Cai F, Kishnani M, Liao XC, Samineni D, Zhu R, Cochran C, Soong W, Diaz JD, Perin P, Tsukayama M, Dimov D, Agache I, Kelsen SG (2016). A randomized trial of the efficacy and safety of quilizumab in adults with inadequately controlled allergic asthma. Respir Res.

[61] Wang Z, Mascarenhas N, Eckmann L, Miyamoto Y, Sun X, Kawakami T, Di Nardo A (2017). Skin microbiome promotes mast cell maturation by triggering stem cell factor production in keratinocytes. J Allergy Clin Immunol.

[62] Nam SY, Kim HY, Kim HM, Jeong HJ (2017). Betaeta-eudesmol reduces stem cell factor-induced mast cell migration. Int Immunopharmacol.

[63] Joulia R, L’Faqihi FE, Valitutti S, Espinosa E (2017). IL-33 fine tunes mast cell degranulation and chemokine production at the single-cell level. J Allergy Clin Immunol.

[64] Zhan M, Zheng W, Jiang Q, Zhao Z, Wang Z, Wang J, Zhang H, He S (2017). Upregulated expression of substance P (SP) and NK1R in eczema and SP-induced mast cell accumulation. Cell Biol Toxicol.

[65] Kritas SK, Caraffa A, Antinolfi P, Saggini A, Pantalone A, Rosati M, Tei M, Speziali A, Saggini R, Pandolfi F, Cerulli G, Conti P (2014). Nerve growth factor interactions with mast cells. Int J Immunopathol Pharmacol.

[66] Berdun S, Rychter J, Vergara P (2015). Effects of nerve growth factor antagonist K252a on peritoneal mast cell degranulation: implications for rat postoperative ileus. Am J Physiol Gastrointest Liver Physiol.

[67] Manning BM, Gruba SM, Meyer AF, Haynes CL (2016). Neuropeptide-induced mast cell degranulation and characterization of signaling modulation in response to IgE conditioning. ACS Chem Biol.

